# Contracting-out urban primary health care in Bangladesh: a qualitative exploration of implementation processes and experience

**DOI:** 10.1186/s12939-018-0805-1

**Published:** 2018-10-05

**Authors:** Rubana Islam, Shahed Hossain, Farzana Bashar, Shaan Muberra Khan, Adel A. S. Sikder, Sifat Shahana Yusuf, Alayne M. Adams

**Affiliations:** 1 School of Public Health & Community Medicine, University of New South Wales (UNSW), Sydney, Australia; 2 Health Systems and Population Sciences Division, International Center for Diarrhoeal Disease Research, Bangladesh (icddr,b), Dhaka, Bangladesh; 3 James P. Grant School of Public Health, BRAC University, Dhaka, Bangladesh; 4 Department of International Health, Georgetown University, Washington DC, USA

**Keywords:** Contracting-out, Primary healthcare, Health systems, Urban health, Non-state actors, Bangladesh

## Abstract

**Background:**

Contracting-out (CO) to non-state providers is used widely to increase access to health care, but it entails many implementation challenges. Using Bangladesh’s two decades of experience with contracting out Urban Primary Health Care (UPHC), this paper identifies contextual, contractual, and actor-related factors that require consideration when implementing CO in Low- and Middle- Income Countries.

**Methods:**

This qualitative case-study is based on 42 in-depth interviews with past and present stakeholders working with the government and the UPHC project, as well as a desk review of key project documents. The Health Policy Triangle framework is utilized to differentiate among multiple intersecting contextual, contractual and actor-related factors that characterize and influence complex implementation processes.

**Results:**

In Bangladesh, the contextual factors, both intrinsic and extrinsic to the health system, deeply impacted the CO process. These included competition with other health projects, public sector reforms, and the broader national level political and bureaucratic environment. Providing free services to the poor and a target to recover cost were two contradictory conditions set out in the contract and were difficult for providers to achieve. In relation to actors, the choice of the executing body led to complications, functionally disempowering local government institutions (cities and municipalities) from managing CO processes, and discouraging integration of CO arrangements into the broader national health system. Politics and power dynamics undermined the ethical selection of project areas. Ultimately, these and other factors weakened the project’s ability to achieve one of its original objectives: to decentralize management responsibilities and develop municipal capacity in managing contracts.

**Conclusions:**

This study calls attention to factors that need to be addressed to successfully implement CO projects, both in Bangladesh and similar countries. Country ownership is crucial for adapting and integrating CO in national health systems. Concurrent processes must be ensured to develop local CO capacity. CO modalities must be adaptable and responsive to changing context, while operating within an agreed-upon and appropriate legal framework with a strong ethical foundation.

## Background

Contracting-out (CO) to non-state providers has been gaining traction as a means of health service delivery improvement in Low- and- Middle- Income Countries (LMICs) [1, 2]. Experience in the health space indicates that the success of CO is largely a function of particular design features and the context in which it is implemented [3]. Loevinsohn & Harding [4] have argued that in developing countries contracting-out to non-state providers (NSP) results in better outcomes than government provision of services. Various studies support this position by demonstrating how collaboration between public and non-state actors, under formal and well-designed contracts, can improve health systems’ capacity and efficiency. In these cases, contracting-out of health care service provision allows sharing of human, financial and physical resources, while reducing duplication of services [5–8]. Open competition and performance incentives inherent in CO arrangements are also thought to motivate providers and improve service delivery [9].

While CO processes are intended to increase access to affordable, cost-effective, and quality services, many implementation challenges exist. These include high administrative costs, unpredictable markets, and lack of government capacity to manage contracts [2, 9]. Defining the precise terms of a contract, while leaving space for contingencies, is an essential capability for a government interested in CO. The financial management capacity of the government is also important. In a weak public system, extra costs will be incurred for external technical assistance and third-party monitoring. Therefore, a closer look at CO implementation processes is needed to identify facilitating factors and potential obstacles. To enhance CO success, in terms of coverage, quality and affordability, it is further necessary to understand how these factors can be addressed in CO design. The discourse on CO for health services improvement has recently explored many such factors, shedding light on implementation of CO in diverse settings and addressing its inherent complexities [10, 11].

In Bangladesh, CO was initiated with international donor support in the late 1990s to bridge the gap in primary health services for the urban poor. This case study of two decades of CO experience in urban Bangladesh aims to add to the global body of evidence by identifying the contextual, contractual, and actor-related factors that positively and negatively influenced the evolution and implementation of contracting-out. This analysis, guided by the Health Policy Triangle framework [12], offers lessons about how the CO approach should respond and adapt to unique and complex circumstances, and what must be anticipated and engaged to enable success. This analysis can inform future plans for CO in Bangladesh, as well as in similar country settings, so that health service provision is responsive, affordable, and accountable to the populations served.

### Setting: The Bangladesh health system

In Bangladesh, the premise that health is a basic human right is reflected in the Government’s constitutional obligation to provide health care services to all citizens [13]. The Ministry of Health and Family Welfare (MoH&FW, or MoH)^1^ is responsible for health policy formulation, regulation, and administration, as well as provision of secondary and tertiary health care at the national level and primary health care (PHC) in rural areas [14]. However, preventive and primary health care provision in urban areas is the responsibility of municipalities and City Corporations (CC), referred to as Local Government Institutions (LGIs), which fall under the Ministry of Local Government, Rural Development and Cooperatives (MoLGRD&Co, or MoLG)^1^ [15–17].

Given increasing demand for health care associated with rapid urbanization, the lack of capacity of LGIs to provide health services effectively has become increasingly apparent. Apart from two large donor funded projects, across urban Bangladesh LGIs operate only a few small- and medium-sized hospitals and outdoor facilities (known as urban dispensaries) that only provide out-patient services [18]. For the urban poor, the lack of PHC services is particularly egregious and results in profoundly inequitable health outcomes. For example in 2013/2014, the Infant Mortality Rate, which in urban areas overall is 34 per 1000 live births and 40 in rural areas, rises to almost 70 in urban slum areas [19, 20]. This pattern also holds for the Under-5 Mortality Rate and the Maternal Mortality Ratio.

Given the paucity of PHC services accessible to the urban poor and the apparent lack of capacity among LGIs to provide these services, a contracting-out mechanism was proposed as a way forward by the Asian Development Bank (ADB). In 1998, the Urban Primary Health Care Project (UPHCP) was established with a loan from the ADB and contributions from other donors [21]. Its specific objectives were to: 1) improve the health of the urban poor and reduce preventable mortality and morbidity, especially among women and children, by increasing access to PHC services; and 2) sustain improvements in PHC by building the capacity of local governments to manage, finance, plan, evaluate and co-ordinate health services [22]. One key component of the project was strengthening institutional governance to sustainably deliver urban PHC services; this was supposed to be accomplished in phase two through developing an operational plan for national urban health and funding coordination with the MoH [23]. In its third iteration, initiated in 2013, the project was renamed the Urban Primary Health Care Service Delivery Project (UPHCSDP).

## Methods

This qualitative study of the implementation processes and experiences undergirding CO in urban Bangladesh was conducted between November 2015 and April 2017. A case study format was employed to generate understanding of complex issues through detailed contextual analysis [24]. To identify key factors, and the intricate relationships among these factors that influence processes with long time horizons (such as the implementation of UPHCSDP), data collection and analysis for the case study were guided by the Health Policy Triangle proposed by Walt and Gilson [12]. This framework highlights four components: context, content, actors, and process. The interactions among these components shape a policy process [12]. Table 1 illustrates the operationalization of the Health Policy Triangle in this study [25, 26].

**Table 1:** Theoretical concepts & their meaning for this study

*Context* can be political, economic and social, at LGI, national and international levels [25]. The politico-economic and social context where UPHCSDP was conceived and implemented also played a major role in supporting and hampering its roll out and eventually to the overall outcome and impact. Both global and national contexts were delved into and attributes external and integral to the health system were separately regarded.	
*Actors* refer to individuals, groups, or organizations who influence the CO approach and its implementation via beliefs, expectations, and position in power structure [25]. Initially, individuals, groups of individuals or organizations, and governments were considered as actors. As our analysis proceeded, to explore the role of actors at several tiers, we grouped them into international, national, local (government, project level), public health providers and health personnel. Their involvement, interest and opposition to UPHCSDP were the aspects of our inquiry.	
*Content* of a health policy, or in this instance, the CO contract, is a reflection of contextual factors and constellation of actors involved, and their interests or ideologies [25]. It provides the basis for implementation and monitoring of the contract’s success. This study therefore captures changes in the content of partnership contracts across the three phases and what influenced these changes; not issues of policy effectiveness and impact [26].	
*Process* is comprised of a range of activities starting from policy initiation, development/ formulation, negotiation, communication, implementation, and evaluation [25]. In this study, we were interested in the effect of the first three concepts on UPHCSDP implementation process.	

### Data collection, sources and tools

Data collection primarily consisted of Key Informant Interviews (KIIs) conducted with past and present stakeholders, including representatives of government, donors, NGOs, and project staff who were or are involved in the design, initiation and/or implementation of one or more phases of UPHCSDP (see Table 2 for categories of respondents).

**Table 2:** Respondent Categories and Number

Respondent Category *(Code)*	Number
Donor (*DNR*)	5
Contract Designer (*CDG*)	3
Ministry of Health (*GOB*)	4
Project staff in PMU, PIU (*PRL*)	12
NGO Head/Manager (*NGM*)	11
Clinic medical officers (*HPN*)	7
Total	42

Conditions for interview were consent to participate (see Declarations for details) and involvement in the project for more than six months. Purposive sampling was initially employed, with snowball sampling used to locate additional KIs involved in the various phases of the project. KIIs were conducted face-to-face using semi-structured guidelines designed to cover the key dimensions of the Health Policy Triangle (see Table 3) [12]. A number of pretests were performed to establish tool validity prior to the initiation of data collection. Data saturation was reached after 42 interviews.

**Table 3:** Major Topics Explored in the Interviews

Topic guides	Health Policy Triangle Dimension			
	Context	Content	Process	Actors
1. Informant’s nature of involvement in the project and duration of involvement				X
2. Need for contracting-out for urban health systems	X			
3. Initial design of the project		X		
4. Steps taken to initiate the project			X	
5. Change over time across the three phases		X	X	X
6. Responsiveness of design changes to the challenges faced			X	
7. Strengths/weaknesses of CO implementation	X	X	X	X

Document reviews were also carried out to provide information on the background of the project, to track contractual and procedural changes over the phases of the project, and to review recommendations made in monitoring and assessment reports. Among the documents considered were project proposals, contract agreements, donor reports, evaluation reports, project documents, program logframes, and other published documents on the UPHCP/UPHCSDP in Bangladesh. The websites of six institutions were searched to obtain the documents: UPHCSDP, the Government of Bangladesh’s Legislative and Parliament Affairs Division, the ADB, the UK Department of International Development (DFID), the Nordic Development Fund (NDF), and ORBIS. Hardcopy documents were retrieved from relevant offices when unavailable in digital format. The information from the documents was used to triangulate interview findings.

### Data management, analysis and validation

Interviews were conducted in Bangla. When the respondent agreed, the interview was digitally recorded; otherwise, verbatim notes were taken in Bangla. All interviews were transcribed and translated into English. A lengthy process of data familiarization occurred before coding was initiated. Prior to data collection, a codebook defining a priori codes was developed drawing from the policy triangle and related CO literature. The codebook was subsequently refined and expanded over the course of the study. Transcripts were coded using ATLAS.ti. Sub-codes were identified in advance (i.e. sustainability, barriers & challenges of finance, staff recruitment & retention, etc.) and inductive codes emerging from the transcripts were defined and applied as the analysis proceeded. For the first 20 interviews, inter-coder reliability was checked by individual coding by two researchers and subsequently comparing the codes. Project documents were also coded using the a priori codes used for coding the KII transcripts or summaries. This facilitated cross-checking and comparison among data sources. To examine interview data, the Framework Analysis Method, in which data displays are created to identify and explore patterns and themes in a systematic manner, was employed [27]. Data displays were analyzed collaboratively by several members of the research team, and analytic memos developed. Respondent validation of study findings was carried out with nine KIs.

### Limitations

Certain methodological weaknesses are acknowledged. Since it was a retrospective study and respondents were asked to recollect events occurring up to twenty years in the past, there is much room for recall bias. Several potential KIs did not respond to the interview request; the most common reason for declining an interview was an embargo by the Project Management Unit (PMU) from communicating with researchers. In addition, the ADB personnel overseeing the UPHCSDP project were inaccessible, leaving the researchers to rely only on interviews with past and present project consultants for that institution’s views. However, notwithstanding the barriers encountered, the rich array of information from various stakeholders and documents lends credibility to the study’s findings.

## Findings

Findings are presented in three sections. To provide some historical context to CO in Bangladesh, the first section briefly describes the 19-year evolution from the UPHCP to UPHCSDP as reflected in changes in the content of the contract. The second section considers the factors that drove the initiation of the project. The third and main section uses the Health Policy Triangle framework to explore the implications of changes in content on the CO process and discusses critical factors driving implementation. This analysis identifies key considerations in play when designing and delivering CO systems for health services in LMICs.

### Evolution of the UPHCP/UPHCSDP

In 1998 the MoLG, with the assistance of the ADB and other donors, launched the Urban Primary Health Care Project to contract-out to NGOs the provision of PHC services for the urban poor. The MoLG has continued to serve as the executing agency of the project; currently a Project Management Unit (PMU) within the Ministry provides technical, administrative, and logistical leadership for project implementation. The project has been implemented continuously in three phases: i) Urban Primary Health Care Project (UPHCP) from 1998 to 2005; ii) Urban Primary Health Care Project II (UPHCP II) from 2005 to 2011; and iii) Urban Primary Health Care Service Delivery Project (UPHCSDP) from 2012 to 2017. A fourth phase of the project was initiated in mid-2017 but implementation had not yet commenced at the time of writing.

Over the three phases, project coverage expanded from four large CCs with a total catchment population of about nine million, to 13 urban centers including smaller municipalities and a ten million catchment population. The project has been marked by constant change in the domains of the Health Policy Triangle. To begin with, the project’s administrative structure changed over time. In the first phase, the project was governed by the Project Implementation Unit (PIU) based at a Local Government Institutions. In the second and third phases, a separate entity called the Project Management Unit (PMU) was formed to monitor, manage and oversee the project under the direction of a senior appointee within the MoLG.

The services stipulated in the CO contract expanded, from an Essential Service Package in the first phase to a more comprehensive package in the latter two phases (Table 4). In terms of remuneration, staff salaries increased in the third phase, but other financial and non-financial incentives diminished. Several respondents noted that the PMU’s follow-through on contracts was lacking, such as failure to disburse performance bonuses for high-performing NGOs in phases two and three. New rules were introduced periodically to guide financial mechanisms and transactions such as a bank guarantee and startup funds (or “mobilization advance”).

**Table 4:** Changes in the content of the contract

Content	Phase 1	Phase 2	Phase 3
Services	ESP Reproductive health care, Child health care Communicable Disease Control Limited Curative Care BCC All national initiatives (e.g. national immunization days, Vitamin A Capsule Distribution, etc.) of the MoH will be supported by the Partnership Agreement	ESD+ Reproductive health care including assistance for women survivors of violence Child health care; Control of communicable disease (tuberculosis, malaria, dengue fever); Limited curative care and first aid for emergency medical care and the treatment of minor infections, BCC, HIV/AIDS, VCT, RTI/STI	ESD+ Basic and comprehensive emergency obstetric care (EOC) facilities, delivering an ESD+ package (including intensive supply of FP logistics, supplementary nutritional support), MCH, adolescent RH care, FP Health promotion and social empowerment activities will be supported through community health worker
Salary structure	Not documented	Gratuity and provident fund along with salary and festive bonus	Only gratuity, No additional benefit
Capacity development of medical officers	1 year full time residential training on EOC & anesthesia		Excluded
Bid security	Bank ID only	Bid security money of BDT 2,500,000	Bid security, as stipulated in the bidding document^a^
Guarantee	Individual performance guarantee	Bank guarantee 10% of contracted budget	Bank guarantee 10% of contracted budget
Mobilization advance (start up fund)- 10% of contracted budget	Deducted partially over several months	Deducted partially with quarterly bills from the first quarter	Deducted at a rate of 16.67%, in the final year and a half of the project

^a^Varied by partnership areas

Source: [25, 36, 38, 62]

The constellation of funders supporting the project changed over time, as did the total budget of the project. The highest allocation was USD 91 million in the second phase (Table 5). The proportion of funds contributed by the Bangladesh government declined, from 25.8% of the total budget in the first phase to 11.5% in the third phase.

**Table 5:** Change in level and source of funding over the three phases of UPHCP/UPHCSDP

Source	Amount in Million USD (Percentage)		
	Phase 1	Phase 2	Phase 3
ADB (loan)	40 (66.7)	30 (32.9)	49.9 (60.6)
ADB (grant for HIV/AIDS)	–	10 (10.9)	–
NDF (grant)	3.5 (5.8)		–
DFID (grant)	–	25 (27.6)	–
SIDA (grant)	–	5 (5.5)	20 (24.3)
UNFPA (parallel grant)	1 (1.7)	2 (2.2)	3 (3.6)
ORBIS (grant)	–	1 (1.1)	–
Government of Bangladesh (GoB)^a^	15.5 (25.8)	18 (19.8)	9.5 (11.5)
**Grand total**	**60.0**	**91.0**	**82.4**

^a^Total contribution of GoB in all phases was 18.42% of the total budget

Source: [23, 28, 29, 36]

One crucial development in the project concerned the bidding process. In phases one and two, technically strong bids were reviewed initially, before the financial component was assessed [23, 28, 29]. As stipulated by ADB procurement rules [23], in the third phase a low-cost bidding system was introduced in which all technical proposals that passed the evaluation were then scored for financial proposal and the lowest bidder received the highest score. The scores of the technical proposals were disregarded in the final stage, resulting in those with the lowest bid receiving the contracts irrespective of their technical proposal scores.

### Factors influencing the initiation of the UPHCP

**Fig. 1:**
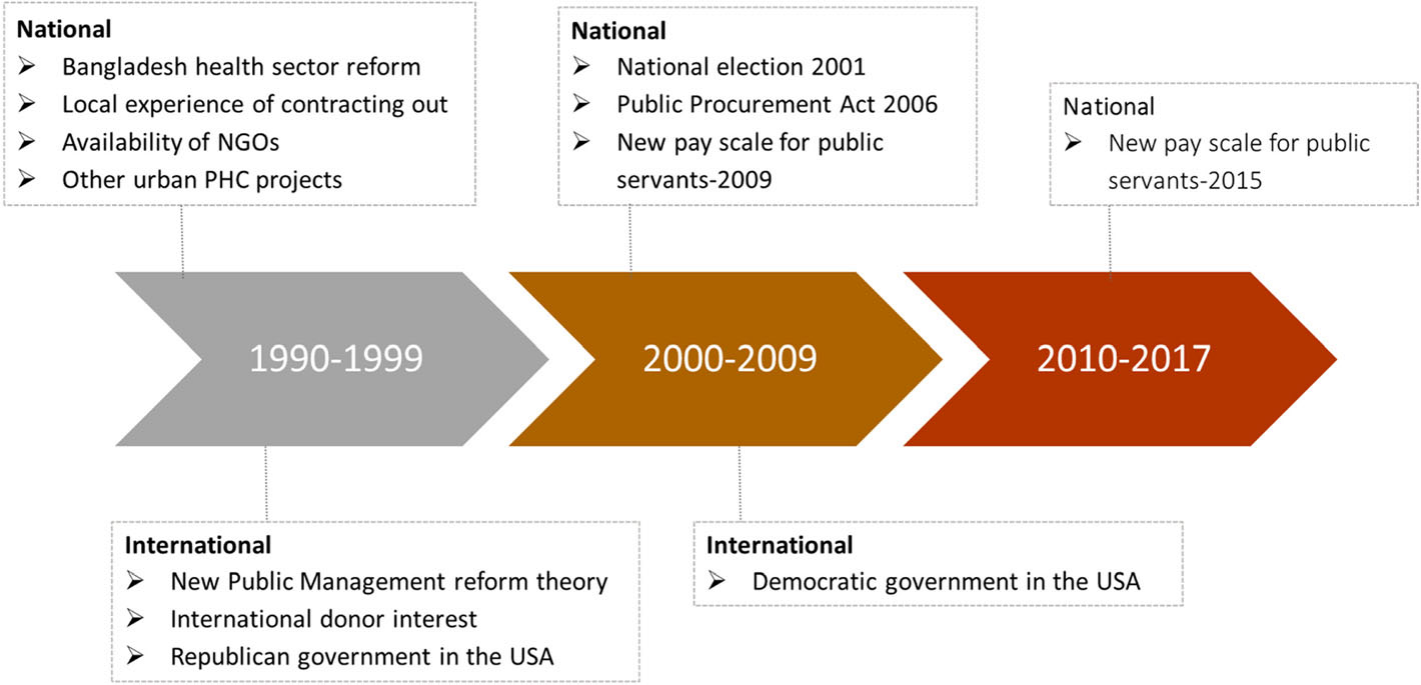
National and international context influencing inception of Contracting-out and driving changes in implementation

Several factors at the national and international levels facilitated the inception of the UPHCP in 1998 (Fig. 1). These ranged from philosophical shifts regarding a government’s responsibilities, stimulated by international financial institutions (NGM-04, PRL-01, CDG-01), to the recognition of existing health system gaps (GOB-01, PRL-01, CDG-01, CDG-03), as well as prior experience with contracting out (NGM-04, PRL-01, CDG-01). The country was also undergoing a health sector reform at that time, which enabled the exploration of new models of service delivery (GOB-04).

Reaching an understanding among the donor agencies involved in the health sector was crucial to forming a funding coalition to support the UPHCP. ADB took the lead, and was joined by the NDF and the UNFPA, both of which shared a common mission of health improvement in LMICs. Not all agreements were documented formally, as noted in one interview:

Two parallel programs funded by the World Bank (WB) were being implemented in Bangladesh [during the ‘90s]. One program was on HIV another one was on nutrition. Then ADB informed that they are also interested to work in health. Now, it is difficult for two strong players to survive in the same field. At that time, an informal mutual understanding was made among the donors: ADB will work in Urban [PHC] and WB will work with Health [national level health care i.e. all of rural and tertiary health care in urban]. (GOB-04)

The MoH’s willingness to sign an agreement with the MoLG, indicating the latter was the executing body of the UPHCP, was also critical (GOB-4, DNR-04). The country’s large and vibrant NGO sector was another factor that made contracting-out viable and enabled market competition among prospective providers. A local champion, whose commitment to the idea of the UPHCP helped dispel initial reticence from the Executive Committee for National Economic Council (ECNEC),^2^ was also centrally important. As one respondent explained:

[The champion] helped to overcome resistance from the government side and from bureaucrats who lobbied against it. But at the end, all of them agreed [to start] the project. (NGM-01)

### Implementation of UPHCP/UPHCSDP

This analysis focuses on the identification of factors that facilitated or hindered project implementation. These are discussed according to the four domains of the Health Policy Triangle -- context, actors, content and process -- with due recognition of the substantial interactions among them.

### Context

#### Service competition

Urban areas are characterized by pluralism and density of health service provision. Failure to take this into account created barriers to project roll-out in the first phase. The initial plans sought to implement UPHCP in all 90 wards of Dhaka City Corporation (DCC). A similar health project called *Shurjer Hashi*, with funding from USAID and in collaboration with the MoH, was already functioning in 38 wards. The KIs generally agreed that negotiations with USAID to avoid overlaps delayed the implementation of UPHCP by a year (PRL-01, PRL-09, NGM-08, GOB-03, NGM-05).

When the project was designed initially, *Shurjer Hashi* was not considered. As *Shurjer Hashi* was a strong player, they said that ‘we are here, we are working, and will continue working.’ (PRL-01)

#### Public sector reform

Other external challenges arose from national level changes in public administration outside the health sector. Pay scale reforms for government service providers, including public doctors, occurred in 2009 and 2015. While the government pay scale for doctors increased, project salaries remained unchanged due to a pre-determined ceiling specified in the contract [30]. Many respondents noted the exodus of doctors from the contracted NGOs as government positions became more lucrative (NGM-02, NGM-07, NGM-09, PRL-06). One participant explained:

People always think that Government service is better…When the Government calls for service, all the doctors and paramedics rush to join…There is no [binding] contract… [with the project and] even with a [signed] contract…they will [definitely] leave. (NGM-02)

Project management faced this problem twice. Proposals for extra funding were required to adjust to this kind of exogenous change (NGM-07, NGM-09, PRL-10). The first time there was no process to adjust for salary increments; however, the second time, in phase 3, the PMU matched the improved pay scale for public services. This decision was a function of learning from implementation challenges in the previous phase, and responding with modifications that allowed these challenges to be avoided or overcome (PRL-03, PRL-04, NGM-07).

#### National politics

Within a few years of the initiation of the UPHCP, a general election took place that resulted in a change in the ruling political party. This brought with it new players with new interests and ideologies. According to some KIs, attendant cronyism and concerns for personal gain and power substantially impacted project implementation. A respondent related their impressions of that period:

[In 2002] Party X came in power. They thought that people working in the project were Party Y [opposition] supporters and…took many new employees [replacing old one]. Then, a consultant [from Party X] was appointed. […] While he was supposed to be a project implementation specialist...he did not do any important work…he politicized the situation. (PRL-01)

This politicization of the project brought about a major change in project administration discussed in the following section.

### Actors

#### Donors

All the donor and funding agencies involved in UPHCP included health improvement in LMICs in their organizational missions. For example, sexual and reproductive health, a key service component in all phases of the project, figures prominently in the mission statements of UNFPA, SIDA and DFID [31, 32]. However, some donors’ missions also resulted in their disengagement over time. The Nordic Development Fund, for example, withdrew following a change in mission to focus on climate change [33]. Similarly, ORBIS, which works solely in the arena of eye care and vision, only collaborated in the second phase when eye care was a component of the project. ORBIS pulled out in the third phase, due to reported internal funding and administrative issues (PRL-04, GOB-04, NGM-07), and the eye care component of the project was subsequently dropped.

DFID’s decision to pull out of the funding coalition in the third phase caused the most disruption. This was especially the case because DFID’s independent evaluation of the project had not revealed substantial weaknesses in project performance [34]. Rather, the decision to withdraw was, according to many respondents, a result of fundamental disagreements about the appropriate funding mechanism (loan vs. grant) and the associated issues of accountability and donor monitoring of the project (NGM-04, CDG-01, CDG-03).

DFID was also concerned that the donor’s contribution to the project was not properly recognized. This was particularly concerning given that it was provided as a grant:

After the second phase, repeatedly we were telling [the Government], “You do not give us [DFID] importance. We gave 28 million pounds or something like this, in dollars it was near 40 million. […] Though the amount from ADB was greater…it was a loan.” (NGM-04)

That is, DFID felt that their concerns about accountability were insufficiently addressed when support was provided as a grant that did not require repayment. DFID’s dissatisfaction is apparent in the evaluation report, which stated “There was little effort to coordinate with the wider donor community from ADB’s side despite membership in the national Health Consortium.” [34].

#### Choice of MoLG as executing body

The designation of MoLG, instead of MoH, as the executing ministry for the UPHCP was described by a number of respondents as an assumed extension by ADB of the mandate of LGIs to provide urban PHC. (PRL-04, PRL-07, PRL-09). A related key element was ADB’s established working relationship with the MoLG on other development projects (PRL-09, GOB-04, NGM-04). However, the MoLG deals with hundreds of developmental projects that are far bigger in scope and funding than the UPHCSDP, so the CO project was perceived by some as an inconvenience (GOB-04, PRL-03). The lack of expertise and interest in health was reflected in minimal MoLG participation in project meetings. Many respondents argued that if the MoH had taken a greater stewardship role, the project would have had a greater chance of eventually being assimilated into the national health system (GOB-02, CDG-01, NGM-01).

#### Politics and power

According to many informants, political motivations and rivalries between actors influenced decisions regarding the inclusion and exclusion of Project Areas (PA) and the engagement of Municipalities and CCs as service providers. Contracts with the Chittagong City Corporation (CCC), the second largest city in Bangladesh, in the first phase and the Gopalganj Municipality, a small but politically influential district and hometown of the leader of the ruling party, in the third phase represented striking deviations from the overall project approach of contracting non-state actors as service providers. In the case of CCC, the Mayor at the time, who successfully negotiated with the project management to receive service contracts, was an influential member of the ruling party. The project management reportedly regarded this as an opportunity to experiment with this modality, agreeing to let CCC cover designated zones in the CC and an NGO (selected through bidding) to serve the rest. However, evaluations comparing service areas found that health indicators in CCC-run facilities fared poorly compared to NGO–supported areas [35]. One respondent suggested these results led to the decision to discontinue the contract with CCC in the third phase.

In Gopalganj, the Municipality was obliged to engage as a direct provider of services because NGOs were unwilling to work in that area, reportedly owing to its reputation as a stronghold of the ruling party. Further, selected NGOs had been unable to provide essential services within the low budget they tendered for and eventually quit the project. This further justified the municipality’s involvement in a direct service provider.

In a number of other instances, it was reported that some PAs were excluded for political reasons. For example, it was suggested by some respondents that the initial inclusion of Narsingdi and Bogra was due to their support for the ruling political party. However, when the government changed parties, those PAs were dropped from the project. This had negative implications for service coverage.

They neither assess the demand nor analyse the supply. […] The centres in Narsingdi and Bogra, have been closed, because these two [partnership] areas have been fixed politically. (NGM-02)

What a waste. Now, [Bogra CC] cannot contract-out that infrastructure or allow another NGO to use it for service provision. (CDG-01)

#### Corruption

Despite the formation of a multi-actor bidding regulation committee to oversee bidding transparency, political favoritism was alleged to have also seeped into the selection of NGOs. According to several respondents, in certain instances the selection of NGOs was reportedly influenced by links to the ruling party; in others, NGOs were reportedly dropped because of their failure to pay “unofficial monies” (CDG-03, NGM-02).

If you look at phase two bidding process, and performance, either NGO A was number one or NGO B was number one […] but they were dropped in phase three […] because they refused, to pay anything. (CDG-03)

One respondent clarified that “unofficial” practices did not occur in all LGIs, and that some LGIs truly valued NGOs’ performance in the previous phase(s) (GOB-03). Another respondent completely dismissed the accusations of corruption, stating they were baseless claims that were “sour grapes” from NGOs that had failed to secure a contract (PRL-09).

#### Locus of leadership

Project leadership shifted during the course of the project due to contextual factors, leading to a corresponding deviation from the contracting-out objective. In the first phase, there was dissatisfaction among CC officials of Rajshahi, Chittagong, and Khulna over the selection of a Project Director from DCC (PRL-09, PRL-12). As discussed in the section on Context, according to our interview respondents, in the second phase an implementation specialist from the newly-elected government was appointed. Political party-backed interests were given precedence over the project’s operation at this time, and the lower ranking Project Director (a chief health officer from DCC) could not overturn those decisions (PRL-01, PRL-09). These realities prompted the ADB to involve the LGD directly and their higher ranked officials instead of working with only the LGIs. Thus for subsequent project phases project administration and all financial responsibilities were transferred from the LGI to the LGD (PRL-01, PRL-09, NGM-01).

This instance clearly demonstrates how a contextual factor – the national election – changed the thrust of the project by influencing actors who in turn disrupted administrative structures and processes. This had other repercussions. With managerial power going to the LGD and its personnel, only the PIU remained within the CCs. The CCs and municipalities became mere implementers reporting to a centralized PMU at the LGD. In so doing, the project’s original commitment to strengthen the managerial and financial capacity of local government was essentially sidelined. To some, this “destroy(ed) the soul of the program” (CDG-02). This weakness was later identified by evaluations and in project documents [28, 34, 36], and was noted by KIs (CDG-02, CDG-03, PRL-01). As one interviewee reflected:

The PIU never really had a great deal of authority. The [original] intention was to devolve responsibilities to them, [and] eventually...contract out to them.…I think the PMU retained and still retains a great deal of the authority. (CDG-02)

### Content

#### Donor influence and restrictions

While most essential services have remained unchanged across the project’s phases, certain services were contingent on particular donors’ strategic interests or constraints rather than the needs of the recipient country. Concerns about this tendency were expressed by one respondent:

HIV is highlighted…but the people are more vulnerable to Hepatitis B than HIV. The agenda of donors, funding opportunities for projects, and easy disbursement of funds…these are important issues to consider. (NGM-02)

The withdrawal of ORBIS before phase 3 meant that eye care was no longer a focus. Similarly, the cessation of grant money for HIV from ADB meant that HIV services were no longer emphasized.

Content is also defined by external influences related to political change and exigency. The “global gag order” imposed by the United States government, which bans financial support to institutions offering or educating about abortion services [37], provides an example. Initially, the restriction of such services was a pre-requisite of UNFPA funding, which originated from the U.S.A [38]. When the gag order was lifted under the Obama administration, permission to conduct “menstrual regulation” by contracted service providers was allowed when medically indicated [30].

#### NGO representation

While the LGIs were chiefly responsible for developing contract documents, with assistance from an agreement specialist or project preparatory technical assistance consultant, inputs from NGOs and other stakeholders on the content of the contract document were also supposed to be included [23, 29, 36]. The extent to which this actually occurred, and the weight given to their inputs, is unclear. But as mentioned above, donor requirements mostly guided the service content.

#### Conflicting service targets

The terms and conditions of the contracts require partner NGOs to provide 30% of their services for free to “the poor, ultra-poor, and at-risk populations.” At the same time, they were given a “conflicting” (PRL-10) target: cost-recovery. This was intended to promote sustainability of the project once donor funding was phased out. The cost recovery targets set specific income generation goals for the NGOs that determined how much they would charge clients for specific services. Most respondents agreed that this was contradictory, as NGOs were not able to recover costs because of their requirement to serve 30% of their poor clientele free of charge.

If you want to serve the poor, you cannot fix a target for income. If an income target is fixed, then serving the poor is impossible. [As for] the sustainability issue, it is [also] a conflicting idea. (PRL-10)

One or more respondents noted various consequences likely related to cost recovery, including an increase in the number of caesarean sections, inappropriate diagnostic tests, unnecessary prescriptions, or taking full payment from poor families normally eligible for free clinic services (DNR-01, NGM-09, PRL-10).

### Process

#### Bid assessments

As mentioned, the change in bidding to focus on selecting the lowest-cost proposal ultimately impacted service quality. The contracted NGOs cut spending on supplies, training and salaries to save money and reach cost-recovery goals. As one NGO manager explained:

We are working to [keep afloat] … Now, the NGO will have to subsidize the cost. For example, in place of five pens, we will buy two. We will make arrangements for training with BDT 20,000 (USD 250) instead of BDT 100,000 (USD 1250). In this way, the NGOs are compromising quality of service due to financial constraints. (NGM-11)

#### Procurement

During the second phase of the UPHCP, the GoB introduced new procurement guidelines that define health as a “service.” However, ADB’s procurement guidelines took precedence over GoB’s guidelines for the CO project (PRL-04, DNR-05); this was made a requirement by ADB to improve transparency and timely procurement [23]. Per ADB’s requirements, the PMU was responsible for the purchase of larger items (such as an ultra-sonogram machine, audio-visual equipment, project vehicles, etc.) [30]. Respondents noted, however, that the quality of the assets purchased was sometimes an issue (PRL-03, NGM-09), as were delays in procurement, both of which negatively affected service provision (NGM-01, NGM-02, PRL-10).

#### Financing

With respect to financing decisions, important adaptations were made by the PMU in the later phases. The bank guarantee mentioned in the UPHCSDP history section was instituted in the second phase. While this change was intended to compel increased accountability of NGOs, the NGOs opposed the clause, arguing that the large sum required discouraged smaller NGOs from participating in the bid. The PMU did take steps to relieve some of the financial burdens faced by partner NGOs by introducing the “mobilization advance.” In the first two phases, advanced money was then deducted from the first quarter of the contract. However, repaying the advance from the very start of the project was difficult for many NGOs that had not yet started generating income, while substantial funding was held up as the bank guarantee. Responding to these financial constraints, in the third phase repayment of the mobilization advance was moved to the last 18 months of project period [23] (NGM-06, PRL-04).

The slow pace of financial disbursements was also problematic; delays occurred because clearance was required from multiple levels due to the separation of the PMU and PIU and bills were held back until all claims were verified [23, 30] (NGM-06, NGM-09, PRL-09). In this area too, amendments were made to rectify the slow financial reimbursement processes. Some KIs reported that the PIU can now withhold costs for problematic claims until resolved but reimburse the rest of the bill.

#### Physician retention

Keeping physicians on staff proved to be a challenge at both the management and the NGO levels. In CCs there is some opportunity to move up the career ladder, from Assistant Health Officer up to Chief Health Officer. However, doctors employed by Municipalities have no scope for career growth (PRL-09, PRL-11).

There is one and only one post for a Health Officer. He has no opportunities for promotion. If he serves there for 30 years he will serve in the same post.…For this reason, no one wants to join, or if anyone joins, within a year they leave for a better opportunity. (PRL-09)

Other reported problems contributing to poor staff retention were difficulties due to local politics, safety at the clinics, and frustrations with a sometimes discourteous public (PRL-11).

Among the NGOs, the retention of physicians involved in service provision was similarly problematic. Some respondents noted one strategy to overcome this challenge: hiring doctors from within the locality where NGO clinics are situated with the understanding that they can supplement their income through dual practice in the private sector:

I live nearby with my family. I never want to go outside of this area. I have freedom of work here.…Now, I am done with my [NGO] work and I will go to my clinic. If anything is needed, I will come again for half an hour or one hour. This is the reason I did not quit this job. (HPN-04)

#### Government’s relationships with partner NGOs

While the need for mutual respect between purchaser (government) and provider (NGO) was emphasized by many respondents, this ideal was not always achieved. The perceived authority of government officials resulted in a tendency to regard NGOs merely as contractors hired to do a job, rather than as project partners contributing to the larger goal of achieving primary health coverage in urban areas. This perception was evident when NGOs preferred having donor agencies present for arbitration during feedback meetings (DNR-02). Partner NGOs were frustrated with interference in staff recruitment processes (PRL-06) and daily activities, especially given their experience and expertise in the health service field (CDG-01, GOB-03). One respondent laughed when describing the situation:

You don’t teach your grandmother how to suck eggs! *[Laughing]* Why should a government bureaucrat know more than they [NGOs] do how to deliver family planning services? *[Laughing].* (CDG-02)

## Discussion

The research described herein provides new data on the intricacies of contracting-out health services by identifying key factors influencing the contracting-out process in Bangladesh, both positive and negative. In reality, these influences are rarely separated in silos; rather they interact and intersect with each other, resulting in implementation processes that are complex and dynamic. The following discussion seeks to embrace this complexity with a view to identifying areas where room for improvement remains in programmatic uptake and integration of CO into Bangladesh’s health system. Specifically, we consider factors hindering the integration of the CO project with the national agenda, key issues impeding the fulfilment of project goals, and the need for an ethical grounding for CO processes. Finally, we address how to think about scale-up in the context of Bangladesh’s continuing economic transition.

### Integration of UPHCSDP into mainstream health services

The CO mechanisms that engage non-state actors to fill gaps in the PHC delivery system in urban Bangladesh have remained quite static over the three phases of the project. In this respect, Bangladesh’s CO experience differs substantially from other countries that have reported their experiences. In Guatemala, for example, both contracting-in and contracting-out were tested [39]. In Cambodia, three different models were successively implemented: a mix of external contracting-in and contracting-out, MoH-donor hybrid contracting-in and contracting-out, and uniform internal contracting-in [10]. In contrast, the contracting-out concept in Bangladesh enjoyed uncontested support from all concerned ministries, enhancing its prospects for sustainability and reducing the risk of reform reversal. However, it is equally the case that a lack of critical discourse on the model may have stifled the meaningful engagement of local urban bodies and other ministries in identifying adaptations of the model to better fit the Bangladesh context.

Of particular note are the minimal involvement of the Bangladesh MoH in setting the direction and course of the UPHCSDP, as well as the project’s administrative location in an isolated unit within the MoLG. Multiple factors contributed to the project’s separation from the MoH: tension between ADB and WB; a misinterpretation of the national ordinance for urban primary health; the ADB’s existing close relationship with MoLG; and the MoH’s focus on a nationwide health and nutrition program at the time of the inception of UPHCP. MoH is represented on the project coordination committee but is not accountable for project implementation. According to respondents, this lack of accountability has thwarted opportunities to integrate the project into the country’s national health program.

The importance of meaningful engagement with the MoH is reflected in similar experiences in other LMICs. For example, Chad’s experience with health systems reform through Results Based Financing (RBF) also demonstrated the risk of not locating project ownership within the MoH; in that case the project was ultimately discontinued [40]. In Ghana, a maternal and child health quality improvement intervention that did not involve the health ministry during its design failed to work out a sustainable mechanism for scale-up [41]. Leadership and organizational support are important factors in the successful scale-up of health service innovations. The critical role of deep engagement of the MoH has not been given distinct consideration or incorporated in relevant frameworks [41] even though the most successful health interventions include MoH involvement [42].

At the same time, the MoLG where the project is housed has demonstrated weak ownership. Given its mandate for local development and lack of health expertise, the capacity of MoLG to negotiate effective health service contracts for Bangladesh seems insufficient. Another consequence of the decision to situate the UPHCSDP within the MoLG was the introduction of unnecessary competition for health human resources. This proved to be a persistent challenge for service delivery by the partner NGOs. It appears, however, that this lesson has been learned; in an endeavor to retain project staff, the upcoming fourth phase of the UPHSCDP proposes to provide salaries competitive with the public sector. However, this raises the possibility of other unintended and undesired consequences, which may be seen in the experiences of other LMICs. For instance, one reason that the MoH in Cambodia has opted away from contracting-out was the leaching of staff from the public health system into the more lucrative non-state sector [10]. These concerns strengthen the argument that keeping the UPHSCDP parallel to MoH activities jeopardizes the likelihood of its long-term integration. Remuneration is not the only factor accounting for staff retention problems; addressing staff turnover also requires grappling with the lack of career progression options, unsafe working environments and perceptions of disrespectful treatment from local leaders and patient attendants..

### Translation of contracting-out objectives into practice

Funding agencies such as ADB, in this case, play a central role in terms of financing; further, they can promote long-term project sustainability through applying sound judgement in selecting the executing body. One of the project’s initial objectives was to build the capacity of LGIs to manage, finance, plan, evaluate and coordinate – that is, to govern – health services. However, the funders failed to intervene to avert the increasing centralization of project governance in the PMU. The management experience of administrative cadres did aid in improving processes to accelerate disbursements to providers, and put in place other financial structures that enabled greater financial stability.

Nonetheless, the failure to build capacity among LGIs deviates from the New Public Management norms on which contracting-out is based: devolving managerial responsibility and creating more participatory decision making processes [43]. Despite 19 years of experience with CO in Bangladesh, many LGIs lack confidence in their ability to write and manage contracts, according to many of the informants. This exemplifies a prominent criticism of CO in LMICs [9, 44–47]. There are, however, counter-examples, instances where LMICs have succeeded in developing expertise and capacities to manage contracts. In both Armenia and Cambodia, for example, strong political and technical leadership by the MoH and country ownership have been identified as the key enablers of capacity development [48].

### Project Management

Our findings suggest room for improvement remains in UPHCSDP contract conditions and procurement practices. The demand on NGOs to provide free services while recovering costs has created difficulties for providers, with many cutting costs on essential materials. Conflicting performance targets make them all unattainable [49]; this has myriad negative ramifications.

Another project management problem that negatively affected service provision was delays in centralized procurement processes. In Cambodia, where contracting-out has been successfully scaled-up, providers had total authority over procurement [10]. Indeed, a case study of CO in Cambodia identified ADB procurement rules as a handicap to meeting targets [47]. The negative implications of centralized systems of procurement are not specific to the ADB. For instance, Bangladesh experienced hardships in meeting the WB’s procurement rules in another contracting-out project [47]. These lessons indicate the importance of developing locally relevant and feasible procurement guidelines that can be adhered to beyond project periods.

### Ethics and the purchaser-provider relationship

Two other issues arose in the study that merit attention in any future CO endeavors in Bangladesh. First, new policy tools for improving health systems performance such as CO are not exempt from the broader challenges faced in the country. Allegations of irregularities in procurement and bidding procedures, as well as reports that personal influence compromised the integrity of the assistance area selection, have surfaced with the UPHCSDP. Jayasinghe [50] identified two factors that can determine PA selection, the state of health in the recipient population and extraneous factors (such as accessibility of an area, local antagonism, and threat to worker safety). The extent to which these criteria were used in UPHSCDP could not be confirmed, but in certain PAs, selection reportedly pivoted around perceived political advantage rather than evidence- or need-based considerations. It also remained unclear why some PAs were dropped between one phase and the next. Nor was any guidance found that addressed the use of infrastructure abandoned when the project pulled out, as in Bogra CC. As argued by Jayasinghe [50], ethical considerations are important in selecting or excluding assistance areas in a CO, yet the ethical quagmires associated with these decisions are not adequately discussed in either project documents or the extant literature on contracting-out.

The relationship between purchasers and providers is another topic that requires more attention. Relationships in CO processes are most often discussed in terms of contract formality, performance requirements, payment formality, or trust in case of relational contracts [51–53]. Interestingly, the social aspects of purchaser-provider interaction are rarely touched upon. This study’s findings suggest that a “spirit of partnership” was absent, and that problematic relationships between the PMU and the NGOs in the UPHCSDP deterred NGOs from participation in the project. This seems to arise from government officials treating “contractors” as subordinates. As long as contracted NGOs remain unable to effectively voice their preferences and concerns, fundamental questions remain about how to hold the PMU and the government accountable in case of breach of contract. The failure of the PMU to disburse promised performance bonuses presents a case in point. Further exploration is warranted into the roles of international funding agencies and legal bodies in Bangladesh, and the extent of their support to NGOs in such matters.

Experiences from elsewhere suggest that a congenial relationship is vital for successful contracting [54]. Ideally, transaction costs are reduced as an initial formal contracting style gradually transforms into a relational contracting arrangement. Relational contracting with a select provider group could reduce adversarial relationships present in more commercial models, thus reducing contract negotiation time [51]. Of course, these relationships depend on the actors involved [55]. Purchaser-provider relationships can be improved by “early agreement on the sources of information to use in negotiations; sharing information where possible; purchasers having a clear purchasing strategy that is communicated to all involved in contracting; developing standard terms and conditions; and developing a style of contracting that is co-operative rather than competitive” [51]. In the case of Bangladesh, the UPHCSDP has a purchasing strategy and standard terms of reference; however, modes of information sharing and negotiation are neither clear nor well-practiced. Co-operative contracting should be discussed in future CO designs as a mean to foster a positive purchaser-provider relationship.

### Thinking forward

To stay relevant, CO strategies need to be dynamic and responsive to changing circumstances, be they political, geographic or financial. As Bangladesh slowly but steadily makes its way towards achieving Middle-Income Country status, it faces parallel declines in donor aid for developmental purposes [56]. Unless local philanthropy steps in or government contracting to NGOs is sustained, it is likely that the number of NGOs will decline. For primary health care services this raises serious concerns. The fiscal space for health in Bangladesh’s national budget is narrow, at only 5.2% of the total national budget in 2017–2018. Government expenditure on health, as a percentage of total health expenditure, is one of the lowest in the South East Asia region [57, 58]. The UPHCP/UPHCSDP experience echoes this; according to documents reviewed for this case study, substantial reductions in government contributions to UPHCP/UPHCSDP have occurred, from 26% in the first phase to 12% of the total project budget in phase three [22, 28, 36]. This raises concerns about ongoing commitments to CO. The MoLG has yet to make a routine budget allocation for urban PHC beyond the project period [23, 59]. Shroff et al. [48], examining experiences from ten countries on RBF uptake, identified the absence of domestic funding as a barrier to scale-up of such health financing arrangements. Moreover, many NGOs in UPHCSDP have struggled to meet their cost-recovery targets. These realities raise questions about the sustainability and feasibility of contracting-out NGOs for PHC in Bangladesh, as well as in other LMICs at a similar development juncture.

## Conclusion & Recommendations

This study, having traced Bangladesh’s urban primary health care CO project’s evolution over its three phases, reveals a myriad of factors that interact and shape implementation of contracting-out to NGOs; these include shifting political environment, donor priorities, and conditions in the contract. These findings are particularly germane given current discourse and planning for the fourth phase. We recognize that CO is not a magic bullet to resolve health service gaps in LMICs. However, it can be successful when used strategically and ethically within a complex and dynamic system.

Based on the lessons learned from this research we recommend the following measures for health systems deliberating about the implementation of CO, and propose some adaptations specifically for the Bangladesh country context:

- *Funders must foster greater country ownership and engagement, both of which are essential for effectively contextualizing the CO process and successful programmatic uptake*

Funding agencies can facilitate and concretize country ownership by thoughtfully and carefully selecting principal agents for CO execution. For health-related projects, the MoH needs to be fully involved, even if the funders have other agendas. Getting the MoH fully engaged also counters the perception that CO diverts health resources. With full engagement, the MoH can frame CO as an important mechanism for resource-sharing with the MoLG; this creates a window for better programmatic integration of CO in the health system.

- *In-country capacity, both structural and process, to do contracting-out must be built*

Despite stated intentions, 19 years of the CO project in Bangladesh has insufficiently developed in-country expertise necessary for programmatic uptake and sustained implementation. In order to “graduate” from a donor-supported project to a national-level program, a critical mass of actors with technical capacity for local-level implementation of CO is required [45]. Funding agencies must be willing to provide adequate resources for training on theoretical concepts and practical skills; rigorous monitoring should ensure that the appropriate actors are given these opportunities, notwithstanding political or bureaucratic favoritism. Fostering a complete theoretical and practical understanding of CO enables receiving countries such as Bangladesh to build skills at the local level.

- *Ground contracting-out processes in a strong ethical and legal framework*

Ethical principles need to be the basis for setting contract terms and regulating contracting practices. Public consultation is important [47], especially regarding the development of ground rules such as how contracting sites are selected, what services will be contracted-out, which NSPs will be engaged, and how effective systems and processes for accountability are incorporated. A well-articulated and agreed-upon ethical framework is especially important in the Bangladesh context to overcome a history of corruption and unlawful political interference. An ethical framework offers a touchstone around which funding agencies, civil society, and CO implementers can coalesce to identify best practices and reduce corruption. To this end, Bangladesh may benefit from creating a regular monitoring mechanism by a third-party ombudsperson. Concurrent strengthening of the legal framework would also serve to bolster the rights of NGOs and NSPs, balancing out an asymmetrical power relationship in which government dominates.

- *Foster true partnership among the key actors*

Successful uptake of new policy tools like contracting-out requires more than developing new technical capacities and skills; it necessitates a rethinking about how collaboration and partnership occurs among actors within and outside of government bodies. This is especially pertinent in countries like Bangladesh, where a strong bureaucratic culture exists. To overcome hierarchies that stifle collaboration, government and donors alike should value NSPs for their contribution to the health system and protect space for them to articulate concerns. Providing greater autonomy, as well as listening, to NSPs may reduce unwarranted interference by government in the management of service provision and foster greater innovation in dealing with challenges. Funding agencies can create the conditions for healthy and equal partnerships by linking conditions of collaboration with fund disbursement, while offering business management training to increase administrative capacity.

- *Be flexible and responsive to changing context*

The current CO model in Bangladesh exclusively partners with not-for profit organizations. Yet at present, only between one and 2 % of all health facilities are NGO facilities. The for-profit private sector thoroughly dominates (> 80%) the urban health landscape in Bangladesh [60]. These private sector providers are impervious to fluctuations in donor aid. However, the contracts under the UPHCP/UPHCSDP have been unable to attract this group of private providers into partnership. Indeed, it would need considerable restyling to appeal to them while continuing to pursue the project’s fundamental objective of increasing affordable quality service coverage for poor people. Engaging with the for-profit private sector entails a risk of cost escalation; this has been the case in South Korea and the Philippines, where Fee-for-Service payment mechanisms have been used with the for-profit sector [61]. Other mechanisms shown to contain costs, such as capitation and a global budget, could be considered. In Thailand, for example, capitation payments have been applied with moderate success, although some private hospitals have been deterred from participating in contracts [61]. Since the private for-profit sector is extremely heterogeneous in Bangladesh, various payment mechanisms would have to be tested for each type of provider should CO with this sector be considered. There is scope to learn from countries with experience in contracting the for-profit private sector, and a need to experiment and adapt these approaches to the Bangladesh context.

### Future research

Retrospective studies such as this one provide general lessons regarding contracting-out in Bangladesh and similar settings. However, project-specific implementation research is needed to yield deeper insight on which mechanisms work and which are failing and into how processes can be reoriented to achieve better and more sustainable results. While systematic reviews on contracting-out healthcare have been published in the past decade, the evidence base available was deemed inadequate to draw concrete conclusions about the merits of this approach in terms of impact, cost effectiveness and sustainability [2, 51]. The systematic review of published primary research over the last decade will provide additional insights on performance and impact level outcomes across geographic regions.

## Abbreviations

ADB: Asian Development Bank
AHPSR: Alliance for Health Policy & Systems Research
BDT: Bangladesh Taka
CC: City Corporation
CCC: Chittagong City Corporation
CDG: Contract designer (code)
CO: Contracting out
DCC: Dhaka City Corporation
DFID: Department for International Development of the United Kingdom
DNR: Donor (code)
ESD: Essential Service Delivery
GoB: Government of Bangladesh
HIV: Human Immunodeficiency Virus
HPN: Clinical medical officer (code)
icddr, b: International Centre for Diarrheal Disease Research, Bangladesh
KI: Key Informant
KII: Key Informant Interview
LGD: Local Government Division
LGI: Local Government Institution
LMIC: Low-and-middle income country
MoH or MoH&FW: Ministry of Health and Family Welfare
MoLG or MoLGRD&Co: Ministry of Local Government, Rural Development & Cooperatives
NDF: Nordic Development Fund
NGM: NGO Head/Manager (code)
NGO: Non-Government Organization
NSP: Non-State Provider
PHC: Primary Health Care
PIU: Project Implementation Unit
PMU: Project Management Unit
PRL: Project level staff at PMU/PIU (code)
RBF: Results Based Financing
SIDA: Swedish International Development Agency
UNFPA: United Nations Population Fund
UPHC: Urban Primary Health Care
UPHCP II: Second Urban Primary Health Care Project
UPHCP: Urban Primary Health Care Project
UPHCSDP: Urban Primary Health Care Services Delivery Project
USAID: United States Agency for International Development
USD: United States Dollar
WB: World Bank
WHO: World Health Organization

## Footnotes

1 MoH&FW will be referred to as MoH and MoLGRD&Co as MoLG in this paper.

2 The National Body, headed by the Prime Minister, is the highest political authority for consideration of development activities reflective of long-term national policies and objectives in Bangladesh
